# Genome Sequences of Two Spore-Forming Bacteria Isolated from the Shore of Mono Lake, California

**DOI:** 10.1128/genomeA.01742-16

**Published:** 2017-03-09

**Authors:** Jason M. Thomas, Natsinet Ghebrendrias, Mamta Rawat

**Affiliations:** Department of Biology, California State University, Fresno, Fresno, California, USA

## Abstract

Here, we report the draft genome sequences of two *Bacillus* spore-forming Gram-positive bacteria, isolated from soil on the shore of Mono Lake.

## GENOME ANNOUNCEMENT

Mono Lake is a shallow, saline, and alkaline lake in the eastern Sierras. The southern shore of the lake is lined with tufa towers, which form from the interaction between shoreline calcium-rich springs and carbonate in the lake water (1). Beginning in 1941, water diversions from the lake to the Los Angeles aqueduct lowered its water level, leaving behind basic and hypersaline soil (2). Mono Lake and the surrounding soil in the lakeshore, including the tufa towers, thus harbor extremophiles with the potential to produce enzymes that are active both at a high pH and high salinity. Alkaliphilic bacteria in particular are valuable for industrial purposes, as alkali-stable extracellular proteases and lipases are now added to laundry detergents, and alkali-stable cellulases are employed in biofuel production to convert lignocellulose in agricultural waste (3). Research on alkaliphilic bacteria has also provided insights on adaptations to extreme environments and the plasticity of biological systems. Alkaliphilic bacteria are also used as models to study ion coupling with ATP synthesis and alkaline pH homeostasis, which has resulted in a greater understanding of flagellin-based motility and chemotaxis (4).

Here, we report the genome sequences of two strains, *Bacillus megaterium* MRML4, and* Bacillus pseudofirmus* MRML5, isolated from soil samples collected from the shore of Mono Lake, CA (37°58.726′N, 119°7.721′W). *B. pseudofirmus* MRML5 was isolated from a soil sample adjacent to a tufa tower, while the soil sample from which *B. megaterium* was enriched was not near a tufa tower. Like the Mono Lake water, the pH of the soil slurry for both samples in distilled water was 9.5. For enrichment and isolation of *Bacillus* species, a soil slurry was made with phosphate-buffered saline (PBS) and heated at 70°C for 30 min. Serial dilutions were plated on Trypticase soy agar (pH 9.5) amended with 3% NaCl. Single axenic colonies of each strain were grown in Trypticase soy broth (pH 9.5) amended with 3% NaCl. The cells were harvested by centrifugation at 15,000 rpm for 10 min. DNA was isolated using the Wizard genomic DNA purification kit (Promega) using the protocol for Gram-positive bacteria. Libraries were prepared and then sequenced on an Illumina MiSeq (version 3 2 × 300 base) by the Indiana University Center for Genome Studies as part of a Genome Consortium for Active Teaching NextGen Sequencing Group (GCAT-SEEK) shared run (5). Assembly of the genomes was performed using the A5-MiSeq assembly pipeline (6, 7). The genome of *B. megaterium* MRML4 was assembled into 88 contigs, with a total length of 5,980,094 bp (*N*_50_, 567,181 bp) and 49.3% G+C content. The genome of *B. pseudofirmus* MRML5 was assembled into 84 contigs, with a total length of 5,286,572 bp (*N*_50_, 12,878 bp) and 36.6% G+C content. The genomes were annotated and mapped to the closest neighbor using RAST (8). Genome mining of these two strains may reveal novel enzymes for industrial applications.

### Accession number(s).

The whole-genome shotgun project sequences for *B. megaterium* MRML4 and *B. pseudofirmus* MRML5 have been deposited at DDBJ/ENA/GenBank under the accession numbers MRUF00000000 and MSLR00000000, respectively. The versions described in this paper are MRUF01000000 (MRML4) and MSLR01000000 (MRML5).
